# Periodontal disease: the influence of metabolic syndrome

**DOI:** 10.1186/1743-7075-9-88

**Published:** 2012-09-25

**Authors:** Enrico Marchetti, Annalisa Monaco, Laura Procaccini, Stefano Mummolo, Roberto Gatto, Stefano Tetè, Alberto Baldini, Simona Tecco, Giuseppe Marzo

**Affiliations:** 1 Department of Life, Health and Environmental Sciences, University of L’Aquila, L’Aquila, Italy; 2 Department of Oral Science, Nano and Biotechnology, University of Chieti, Chieti, Italy; 3 Department of Oral Science, University of Tor Vergata, Rome, Italy

**Keywords:** Oxidative stress, Metabolic syndrome, Adipocytokines, Periodontitis

## Abstract

Metabolic syndrome (MetS) is a cluster of cardiovascular risk factors that include obesity, impaired glucose tolerance or diabetes, hyperinsulinemia, hypertension, and dyslipidemia. Recently, more attention has been reserved to the correlation between periodontitis and systemic health. MetS is characterized by oxidative stress, a condition in which the equilibrium between the production and the inactivation of reactive oxygen species (ROS) becomes disrupted. ROS have an essential role in a variety of physiological systems, but under a condition of oxidative stress, they contribute to cellular dysfunction and damage. Oxidative stress may act as a common link to explain the relationship between each component of MetS and periodontitis. All those conditions show increased serum levels of products derived from oxidative damage, promoting a proinflammatory state. Moreover, adipocytokines, produced by the fat cells of fat tissue, might modulate the balance between oxidant and antioxidant activities. An increased caloric intake involves a higher metabolic activity, which results in an increased production of ROS, inducing insulin resistance. At the same time, obese patients require more insulin to maintain blood glucose homeostasis – a state known as hyperinsulinemia, a condition that can evolve into type 2 diabetes. Oxidation products can increase neutrophil adhesion and chemotaxis, thus favoring oxidative damage. Hyperglycemia and an oxidizing state promote the genesis of advanced glycation end-products, which could also be implicated in the degeneration and damage of periodontal tissue. Thus, MetS, the whole of interconnected factors, presents systemic and local manifestations, such as cardiovascular disease and periodontitis, related by a common factor known as oxidative stress.

## Introduction

This review inquires into and tries to clarify the relationship between metabolic syndrome and periodontitis.

Periodontitis is a family of diseases that affect dental supporting tissues, caused by infections sustained by periodontal pathogens such as *Porphyromonas gingivalis, Prevotella intermedia, Tannarella forsythia,* and *Aggregatibacter actinomycetmcomintans*, which lead to soft and hard tissue destruction, dental mobility, and the loss of dental elements [1].

Susceptibility to these diseases is highly variable and depends on host responses to periodontal pathogens. Although bacteria cause plaque-induced inflammatory periodontal disease, the progression and clinical characteristics of these diseases are influenced by both acquired and genetic factors that can modify susceptibility to infection [2].

Periodontitis in the United States has a prevalence of 30% to 50% of the population, but only about 10% have severe forms. It tends to be more common in economically disadvantaged populations or regions. Its occurrence decreases with a higher standard of living. Individuals of Israeli, Yemenite, North-African, South Asian, or Mediterranean origin have a higher prevalence of periodontal disease than individuals from European areas [3].

Periodontitis must be distinguished from gingivitis (properly, inflammation of the gum tissue), which is a term used to describe a non-destructive periodontal disease [4].

The pathophysiological mechanism of gingivitis is in response to bacterial biofilms adhering to tooth surfaces. Epidemiologically, gingivitis is the most common form of periodontal disease. From a prognostic point of view, in the absence of treatment, gingivitis may progress to periodontitis, which is a destructive form of periodontal disease [4], but in some sites or individuals, gingivitis never progresses to periodontitis [5].

Data indicate that periodontitis is always preceded by gingivitis [6], while gingivitis can be prevented through regular oral hygiene that includes daily brushing and flossing [7,8].

Periodontitis depends on host responses to periodontal pathogens.

The initial increased presence of neutrophils at the site is followed by the release of cytokines by neutrophils and macrophages; the chemical mediators released include tumor necrosis factor alpha (TNF-α), interleukin-1 (IL-1), and prostaglandins.

The inflammatory process includes the stimulation of fibroblasts by IL-1 and the secretion of matrix metalloproteinases (MMP), of which collagenase is the most prominent, by polymorphonuclear neutrophils. MMPs are responsible for increased collagen breakdown, and TNF-α is primarily responsible for increased osteoclast activity resulting in bone resorption.

T-lymphocytes secrete receptor activator of nuclear factor kappa-B ligand (RANKL), which is involved in osteoclast activity and, therefore, bone resorption [9].

Periodontitis has also been associated with elevations in circulating levels of IL-6 and C-reactive protein (CRP). IL-6 is an important proinflammatory cytokine involved in the regulation of host response to tissue injury and infection. It is produced by a variety of cells, such as monocytes, fibroblasts, osteoblasts, and vascular endothelial cells, in response to inflammatory challenges. Moreover, it is widely accepted that IL-6 induces CRP production.

In addition, a significant overexpression of IL-21, IL-1β, IL-17, and IL-23p19 has been detected in tissues affected by periodontal disease compared with healthy gingival tissues. In particular, IL-21 is overexpressed in chronic periodontitis gingival tissues and is correlated with the clinical parameters of periodontal destruction and with proinflammatory cytokines [10].

A negative modulatory role of IL-4 and IL-13 in osteotropic cytokine production could be a mechanism that plays an important inhibitory role in inflammation-induced periodontitis. In facts the activation of STAT6 by IL-4 and IL-13, through type 2 IL-4 receptors, seems to inhibit the production of IL-11 and leukemia inhibitory factor stimulated by IL-1β and TNF-α in human gingival fibroblasts [11].

In addition, IL-10 and tumor growth factor-β1 (TGF-β1) are down-regulated in periodontal lesions. Generalized aggressive periodontitis subjects are characterized by a higher IL-1β/IL-10 ratio than are periodontally healthy subjects, suggesting an imbalance between pro- and anti-inflammatory cytokines in generalized aggressive periodontitis. IL-10 is also associated with periodontal health and seems to be a regulator of inflammation and alveolar bone loss in periodontal diseases. It might be involved in controlling the inflammatory process at periodontally healthy sites [11].

## Metabolic syndrome

Metabolic syndrome (MetS) is characterized by multiple disorders. Oxidative stress seems to have a great role in the ethiopathogenesis of MetS and is the common factor useful to explain the interconnection of all the components of MetS.

**Figure 1:**
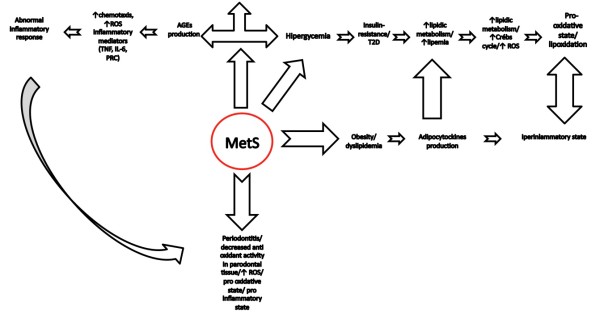
Flow chart that indicates the pathophysiological mechanism underlying the onset of Mets.

In this review we analyze singularly the overlapping factors of each component and the common ways of ethiopathogenesis Figure 1.

MetS is a pathology largely diffused in western countries. Its diagnostic criteria have only recently been better defined, even though they are still ambiguous, because these diseases, including obesity, hyperglycemia, hyperinsulinemia, and dyslipidemia, are all considered serious risk factors for heart diseases [12,13]. As MetS is characterized by numerous factors, it is very difficult to establish a correct diagnosis and an adequate therapy.

MetS was initially defined as Syndrome X by Reaven [12] and then as the “deadly quartet,” referring to the synergy of its components, such as hyperinsulinemia, hypertension, hypertriglyceridemia, and visceral obesity. Subsequently, it was defined as insulin-resistance syndrome, since authors believed that insulin resistance was the dominant factor predisposing the occurrence of other symptoms.

Besides the risk factors already mentioned, other peculiarities that seem to be associated with MetS include hepatic steatosis, inflamed adipose tissue, enhanced clotting factor activity, endothelial dysfunction, inflammation, and obviously oxidative stress [14].

Numerous definitions have been proposed for MetS, but the criteria proposed by the National Cholesterol Education Program (NCEP) Adult Treatment Panel III are actually the most used [15]. The American Heart Association describes MetS as a syndrome characterized by a group of metabolic risk factors in an individual [16]. These factors include the following:

Abdominal obesity, i.e., excessive fat tissue in and around the abdomen;

Atherogenic dyslipidemia, i.e., blood fat disorders, high triglycerides, low high-density lipoprotein cholesterol (HDL-C), and high low-protein lipoprotein cholesterol (LDL-C) that foster plaque buildup in the artery walls;

Elevated blood pressure;

Insulin resistance or glucose intolerance, i.e., the body cannot properly use insulin or blood sugar;

Prothrombotic state, e.g., high fibrinogen or plasminogen activator inhibitor-1 in the blood; and

Proinflammatory state, e.g., elevated CRP in the blood.

The American Heart Association and the National Heart, Lung, and Blood Institute recommend that MetS be identified as the presence of three or more of the following components:

Elevated waist circumference:

Men – equal to or greater than 40 inches (102 cm);

Women – equal to or greater than 35 inches (88 cm);

Elevated triglycerides: equal to or greater than 150 mg/dL;

Reduced HDL (“good”) cholesterol:

Men – less than 40 mg/dL;

Women – less than 50 mg/dL;

Elevated blood pressure: equal to or greater than 130/85 mm Hg;

Elevated fasting glucose: equal to or greater than 100 mg/dL.

However, some authors do not find as clinically useful MetS diagnosis as a defined entity. They consider definition criteria and clinical and laboratory parameters to be ambiguous and incomplete, and the cardiovascular risk factors associated with MetS the same as pathologies considered separately [17]. Moreover, they argue that total therapy does not differ from that for each single disease and that the hypothesis of insulin resistance as a common cause is uncertain [18].

People with MetS are at increased risk of coronary heart disease or other diseases related to plaque buildup in the artery walls, such as stroke and peripheral vascular disease and type 2 diabetes. The preponderant, fundamental risk factors for this syndrome appear to be obesity and insulin resistance, but other conditions associated with the syndrome include physical inactivity, aging, hormonal imbalance, and genetic predisposition as seen in the handicapped and senior citizens.

The necessity of organizing the exact diagnosis of MetS arises from the need to determine patients at extreme risk of developing cardiovascular disease (CVD) and to establish an appropriate therapy and prevention [19]. The first goal of the clinical management of MetS is to reduce the major risks for CVD and type 2 diabetes by stopping smoking, stabilizing LDL and blood pressure parameters, maintaining glucose levels at the recommended values, reducing body weight (body mass index less than 25 kg/m^2^) through an adapted diet, and doing moderate-intensity physical activity for at least 30 minutes on most days of the week.

## Oxidative stress and metabolic syndrome

As previously shown, the role of oxidative stress in the ethiopathogenesis of MetS and its characteristic signs is widely supported [20].

Reactive oxygen species (ROS) are physiologically produced by the cellular metabolism. They are highly reactive species, able to exist independently, and are characterized by the presence of unpaired electrons that allow interaction with a great number of biomolecules, causing their oxidation [17,21]. Exogenous sources of ROS are smoking, ultraviolet light, heat, ultrasound, ozone, radiation, exhaust fumes, infection, excessive exercise, trauma, and drugs, whereas endogenous sources include products of metabolic pathways and products of immune and connective tissue cells [22,23]. Antioxidants are substances that can inhibit the action of oxidant species [21]. There is a physiological fine balance between oxidant activities and antioxidant defenses, but when this equilibrium is disrupted to the advantage of ROS, or to increased ROS activity or to want of antioxidant defenses, the result is oxidative stress [22]. In this condition ROS operate by creating an adequate environment for phagocytic vacuole and enzymatic digestion, and by mediating cellular signaling. An amplified activity of ROS implies a large spectrum of molecular and cellular damage, such as lipoxidation. This results in covalent binding with proteins, which alters their structure and function [22]. Some oxidized proteins are difficult to remove by cells and tend to accumulate with aging and in the presence of chronic diseases such as diabetes mellitus.

Several studies have demonstrated a real correlation between oxidative stress and MetS. In fact, in patients suffering from MetS, systemic oxidative stress seems to be more elevated than in healthy controls, and antioxidant defense seems to be decreased, as demonstrated by the diminished rate of Vit C, α-tochopherol, and superoxide dismutase activity in serum, and by increased lipoxidation [24]. Moreover, obesity is firmly related to oxidative stress–mediated endothelial damage [25]. An increased caloric excess not balanced by an elevated energy expense leads to an increase in the metabolism of Krébs cycle, generating a ROS excess [26]. Some HDL-C subfractions present antioxidant activity that is diminished in people suffering from MetS, and this reduction is correlated with systemic oxidative stress and insulin resistance. Furthermore, obese adults with MetS have an increased plasmatic rate of oxidized LDL-C compared with obese patients without this syndrome [27]. In addition, individuals suffering from MetS present an increased presence of serum lipid peroxide compared with healthy controls [28].

Insulin resistance is a condition in which the normal amount of insulin is insufficient to obtain an adequate response from muscular and adipose tissues and from hepatic cells, and this leads to a severe hyperglycemia with deleterious systemic effects [29-32], such as lower intracellular antioxidant defenses [31,32].

A great number of studies have focused on molecules that can reduce oxidative stress. Recently, for example, some studies have proposed tetracycline as useful in combating oxidative stress in periodontitis and metabolic disorders. In fact, in addition to its antimicrobial effect, it shows antioxidant, anti-inflammatory, proanabolic, immunomodulatory, angiogenetic, and antiapoptotic effects [33,34]. Decreased yields of oxidative stress were also obtained in the presence of minocycline, which demonstrates its potential role as an adjunctive therapeutic agent in an environment of oxidative stress, such as in periodontal disease and coexisting cardiometabolic pathologies [35]. Then, the beneficial systemic effect of the antioxidant tempol on apical periodontitis in both control and pathological rats with doxorubicin-elicited cardiomyopathy has also been evidenced [36]. In addition, a recent study focused on the antioxidant, anti-human immunodeficiency virus, anticarcinogenic, and anti-inflammatory properties of Gomisins G and J extracted from *Schisandra chinensis*, as it seems to inhibit *Porphyromonas gingivalis*[37]. Furthermore, the proanthocyanidins (PAC), the most abundant flavonoids extracted from red cranberry fruits, have been reported to possess antimicrobial, antiadhesion, antioxidant, and anti-inflammatory properties. The PACs have variable pharmacological and nutraceutical benefits, including improvement of ischemic CVD, prevention of atherosclerosis, and antiarthritic, anticancer, and antimicrobial activities. A recent *in vitro* study has shown that cranberry PACs may be also potential therapeutic agents for the prevention and management of periodontitis [38].

Thymoquinone has also demonstrated a variety of pharmacologic properties, including antihistaminic, antibacterial, antihypertensive, hypoglycemic, anti-inflammatory, and antioxidative activities. Through its anti-inflammatory and antioxidant properties, thymoquinone seems to play an important role in preventing periodontal diseases [39].

Aso, S-nitrosoglutathione is a nitric oxide donor that seems to exert antioxidant, anti-inflammatory, and microbicidal actions, and has been demonstrated as a potential drug for the topical treatment of periodontitis [40]. Finally, because of its anti-inflammatory effects, a novel α-iso-cubebenol isolated from the dried fruit of *S. chinensis* is considered a novel therapeutic agent to ameliorate periodontitis [41].

## Hyperglycemia and periodontal diseases

Diabetes, a pathology that is extremely widespread, involves an adulterated homeostasis in the glucose metabolism. Two types of diabetes exist: type 1 diabetes and type 2 diabetes (T2D). Either typology of diabetes, if not controlled, is a risk factor for periodontitis.

The first type affects young people and sets in from childhood. The pathological mechanism is that β cells of Langerhans are unable to produce insulin. Consequently, glucose tends to accumulate in the tissues and the blood. The pathology develops in middle age.

T2D is the most common type and consists in an adulterated insulin cellular response. The onset of T2D is associated with the recruitment of proinflammatory cytokines that are involved in the onset of the disease and related complications such as dyslipidemia and atherosclerosis, contributing to the onset of microvascular and macrovascular complications [42,43].

Furthermore, patients with periodontitis show a higher risk of ketoacidosis, retinopathy, and neuropathy than do diabetic patients without periodontal disease. In addition, diabetic patients with neurological complications have severe gingivitis compared with diabetic individuals without this complication. Many studies demonstrate a biological relation between diabetes and periodontitis, as tested relations exist between glycated hemoglobin, a diabetic marker, and periodontal parameters, and between plasmatic lipid peroxide, an index of oxidative stress, and periodontal markers [44,45].

Regarding the mechanism involved in this association, it seems that prolonged hyperglycemia associated with diabetes causes the formation of advanced glycation end-products (AGE). AGEs are physiologically produced by the organism, but in conditions of hyperglycemia or augmented oxidative stress their presence is largely augmented. Exogenous sources of AGE are smoking and browned foods [46]. AGEs result from reversible glycation reactions, that is, the addition of sugars on the polypeptide chain of proteins, lipids, or nucleic acids. Whether an enzyme attends or not in the glycation reactions, we can have an enzymatic or nonenzymatic glycation [46]. Furthermore, more resistant proteins such as interstitial or vassal collagen can undergo a nonenzymatic glycation. Several cells, such as endothelial, muscular, and immune cells, possess specific AGE receptors, called RAGE. In fact, a confirmation of AGE activity against periodontal tissue is proved by an elevated presence of RAGE in the periodontal compartment [47].

AGEs have deleterious effects for the organism and, being resistant to proteolytic digestion, are difficult to eliminate and tend to accumulate, expressing their harmful effect. AGE-degradation products are chiefly expelled through urine [48].

AGEs are substances able to promote cytokine production by macrophages such as TNF-α and IL-6, and to stimulate hepatic secretion of acute-phase proteins such as CRP, fibrinogen, plasminogen activator/inhibitor, and serum amyloid A, also correlated with oral infection and cardiovascular diseases, especially in patients suffering from periodontal diseases [46,48]. AGEs also promote monocyte migration and increase endothelial permeability, fibroblasts, and muscular cell activity [46]. In addition, AGEs can bind collagen, which if modified alters basement membrane structure. This gives to an inhibition of oxygen diffusion and tissular oxygenation, diminished waste removal, altered immune mediators, and migration that causes an adulterate inflammatory response, which results in inadequate tissue recovery [42].

AGEs also lead to the rapid expansion of energy in the respiratory polymorphonuclear neutrophils (PMN), and this causes increased damage to periodontal tissues [42,49] and some changes in bone metabolism, especially on repair, also reducing the production of the extracellular matrix [50].

The course of the periodontal illness may be due to basic neutrophilic activity that results in an exaggerated immune response and an uncontrolled ROS production. These cause local and peripheral oxidative damage, both directly, through oxidative injury, and indirectly, by means of redox-sensitive gene transcription factors. Nuclear factor kappa B and activating protein 1 are activated, triggering an inflammatory mediator cascade and quick cellular aging [51].

Diabetic patients with severe periodontitis have demonstrated a reduced chemotaxis of PMN compared with healthy subjects, or patients with less aggressive periodontitis, and an altered control of apoptosis, which is followed by an accumulation of PMNs in the periodontal tissues and therefore of MMPs and ROS [50]. The lack of glycemic control has also been associated with alterations in the oral mucosa barrier [52]. The presence of diabetes seems to increase inflammation in the periodontium, so much so that in the gingival crevicular fluid there are increased levels of PGE2 and IL1-β compared with parodontopatic normoglycemic patients. Moreover, the presence of diabetes mellitus prolongs the inflammatory response to *P. gingivalis*, causing an increase in the production of TNF-α [50].

In synthesis, diabetes appears correlated to the onset of periodontal diseases, both through changes in the host immune response and through anomalies in the collagen metabolism. Recently, different molecules have been investigated for the treatment of diabetes.

Decursin (De), an active component of *Angelica gigas*, known to exert anticancer and neuroprotective effects, was recently studied for its antiobesity and antidiabetic potential. De treatment resulted in the inhibition of adipocyte differentiation and expression of fatty acid synthase. Administration of De along with a high-fat diet (HFD) significantly reduced the secretion of HFD-induced adipocytokines such as leptin, resistin, and IL-6 [53].

Periodontal inflammation can later give rise to a systemic inflammatory state. A recent study suggested a concrete relationship between severe periodontitis and systemic oxidative stress with a reduction in antioxidant systemic defenses. Moreover, it seems that after a periodontal treatment, a transitory inflammatory acute response occurs with a concomitant increase of systemic oxidative markers [54]. In periodontal diabetic individuals suffering from T2D, there is an augmented rate of oxidative systemic markers and a diminished plasma small-molecule antioxidant capacity [54]. These patients also show reduced β-cell activity. Therefore, it seems that periodontitis enlarges oxidative stress–mediated dysfunction in β-cells of Langerhans [55]. Even in periodontitis, the mediated response of the host, in the presence of bacterial infections, results in an increased release of systemic inflammatory mediators. The toll-like receptors (TLR), the receptors for the Ig superfamily (TLR4), and RAGE are implicated in the intensification of the systemic immune response during chronic diseases, such as diabetes and periodontal disease [47]. Fibroblasts, macrophages, and epithelial cells present in the periodontium possess both RAGE and TLR4 [47]. It seems that hyperglycemia can promote the expression of these receptors in the periodontal district and increase the response to their ligands, favoring the accumulation of these ligands [47]. Several studies have shown how chemical and mechanical periodontal treatment may improve glycemic and lipidic control in time [56-60]. It seems that periodontal therapy leads to a reduction in the glycated hemoglobin (HbA1c) plasma rate and to a better lipidic profile [59,60].

## Atheroma and periodontitis

Periodontitis also contributes to atherosclerosis and CVD [61].

Periodontal pathogens draw lymphocytes in an attempt to stem infection through phagocytosis and killing, causing augmented production of ROS, which ends in a situation of oxidative stress. The presence of ROS promotes chemotaxis and recruits inflammatory mediators from the liver, causing periodontal destruction and favoring the making of endothelial atheromas.

Periodontal pathogens are also able to invade the endothelium and atheromas; in fact, oral infection, being a source of bacteria, is associated with CVD [62].

There are several theories that attempt to explain the correlation between periodontitis and CVD [63].

One theory is the *bacteriological theory*, according to which oral pathogens, such as *P. gingivalis*, invade the systemic circulation and by means of the virulence factors, such as fimbriae, are able to invade the atheromatous plaques. These bacteria can activate endothelial cells through TLR4 and induce apoptosis in these cells, disrupting the mechanisms of cell adhesion.

The *inflammatory theory* holds that in the course of periodontitis, gingival cells produce inflammatory mediators such as TNF-α, IL-6, PGE-2, and MMPs that locally promote tissue destruction and, once in the circulation, stimulate endothelial cells to produce other mediators such as monocyte chemotactic protein (MCP-1), macrophage colony stimulating factor (MCSF), intercellular adhesion molecule (I-CAM), vascular cell adhesion molecule (V-CAM), P-selectin, and E-selectin. These cytokines accelerate the formation of atheroma.

The third theory is the *autoimmune theory*, according to which antibodies against bacterial antigens may also react against endothelial protein, causing their destruction and therefore the arterial lesion [63].

Systemic and local chronic inflammatory states, such as periodontitis and MetS, are characterized by an elevated presence of acute-phase proteins such as CRP and fibrinogen, which represent a decisive contribution to the insurgence of atherosclerosis and CVD [63]. Vice-versa, the reduction of periodontal inflammation through plaque control, systemic antibiotics, scaling, and root planning seems to decrease CRP levels in patients suffering from MetS, reducing the risk of CVD [63].

## Obesity and periodontal damage

The clear relationship between obesity and periodontitis is well documented [64-92]. Overweight is a clear risk factor for the onset of T2D and CVD, as well as for respiratory and pressure disorders, osteoarthritis, reproductive abnormality, hepatitis, and some types of cancer [64]. Adipocytes of fat tissue show the ability to secrete adipocytokines, which seem to be very important in controlling appetite and body weight. One of these cytokines is leptin, which shows a protective role against obesity [17]. In fact, a condition of clear obesity can be also defined as a situation of leptin resistance with a consequent leptinemia [65].

Adipocytes can secrete other cytokines such as adiponectin and resistin. Serum adiponectin remains constant in normal condition but decreases in the presence of diabetes, obesity, insulin resistance, and CVD [66]. Resistin shows a great proinflammatory role, and from studies conducted on mice it seems associated with insulin resistance [17,22].

However, many researches affirm that the most important mediator related with obesity and insulin resistance is TNF-α, expressed plentifully in adipose tissue, in obese individuals with severe insulin resistance, and in neoplastic patients [67,68]. Both TNF-α and Il-6, secreted by adipose cells, seem to trouble intracellular signaling, cause insulin resistance, and stimulate hepatic production of phase acute-phase proteins such as CRP [69]. Recent murine studies show that the direct infusion of TNF-α or LPS, a TNF-α inductor, can cause a severe insulin resistance; by contrast, mice missing TNF-α or its receptor-codifying gene and submitted to a fatty diet appeared protected against insulin resistance compared with controls in the same condition having genes [70]. It seems that when insulin binds its tissular receptor, a receptor tyrosine residue on the cytoplasmatic portion is autophosphorylated. The tyrosine residue of the cytoplasmatic insulin-receptor substrate (IRS-1) binding insulin receptor is phosphorylated and causes an intracellular signaling, which leads to the membrane recruitment of intracellular transporter of glucose, GLUT-4, to trap glucose molecules. TNF-α seems to be able to phosphorylate a serine residue on IRS-1 and, subsequently, to inhibit phosphorylation of either the insulin receptor or IRS-1.

Phosphorylation on IRS-1 can also prevent IRS-1 Tyr phosphorylation on adipocytes [72].

Other authors have supposed that TNF-α can interact with RNA transcription codifying for IRS-1 and GLUT 4, compromising their stability. It could also prevent Tyr phosphorylation of the insulin receptor, inducing hydrogen peroxide formation [22,72].

Regarding the relationship between periodontitis and obesity, recent studies have demonstrated how normal-weight persons who participate in sports and physical activity show a decreased incidence of periodontal disease [73-76]. Recent studies have also demonstrated how individuals with normal weight showed a lower prevalence of periodontitis, decreased plasma levels of inflammatory markers, and increased insulin sensitivity [77]. It is of note that during the development of periodontitis, in the attempt to remove causal agents, hyperinflammation is triggered, attracting numerous neutrophilic granulocytes, which, through the production of digestive enzymes during phagocytosis and killing, favor the production of ROS and the consequent constant release of inflammatory mediators with direct bacterial action damage on periodontal tissue. In addition, it seems that in people suffering from periodontitis, pathogen bacteria, endotoxins, and inflammatory mediators cause leucocytosis and increased lipidic metabolism, with an increase of cholesterol and hepatic triglycerides favoring the risk of CVD [78,79]. Furthermore, a systemic increase of oxidative stress caused by periodontal disease seems to promote LDL oxidation [80]. LDLs are cholesterol carriers with proinflammatory and pro-atherogenic activity. Periodontal treatment by scaling and root planning, and dental care instruction seem to reduce the circulating oxLDL level and, consequentially, the risk of the onset of CVD [80]. In addition, leptin seems to be involved in bone metabolism [74]. In recent murine studies, animals missing the leptin-codifying gene developed pronounced obesity. At the same time, mice with an adulterated leptin receptor presented overweight and T2D [66]. Moreover, children lacking leptin showed severe obesity, but when exogenous leptin was given, obesity decreased considerably. In periodontal individuals, serum leptin seems increased [81,82]. On the contrary, leptin in gingival fluid shows decreased levels, especially in the presence of aggressive and advanced periodontitis, advising of a protective leptin role, although this topic is still disputed [84].

There are no relevant data about the rate of adiponectin in gingival sulcular fluid, but *in vitro* it has been shown to be able to inhibit osteoclast activity induced by LPS. Thus, it seems to have anti-inflammatory and protective action against the progression of periodontitis and a predictive role for T2D [74].

In synthesis a recent systematic review confirmed a positive association between obesity and periodontal diseases across multiple studies and populations from around the world, but this relationship needs more investigation [84]. The relationship appears clarified when the treatment of obesity is also analyzed. According to a recent study by Lakkis et al. [85] some parodontopathic obese patients who underwent bariatric surgery showed weight loss and fat-mass loss after surgery, and an improvement in inflammatory conditions with the reduction of circulating markers and hyperglycemia. In addition, it seems that patients who undergo bariatric surgery show an improved response to non-surgical periodontal therapy compared with obese parodontopathic patients not treated with bariatric surgery. These data contrast with those reported by a study conducted by Zouza et al. [86], who showed that non-surgical periodontal treatment allows a reduction of all clinical parameters of periodontal inflammation, both in obese patients and in normal-weight subjects, arguing that obesity does not negatively affect the success of periodontal therapy. Some studies also try to magnify this relationship. A recent meta-analysis indicated statistically significant associations between periodontitis and body mass index: the category of obese subjects showed an odds ratio (OR) of 1.81 the category of overweight subjects showed an OR of 1.27, and obese and overweight combined showed an OR of 2.13. Although there is insufficient evidence to provide guidelines to clinicians on the clinical management of periodontitis in overweight and obese individuals [87], these studies evidence a clear relationship.

Bariatric surgical procedures, including the laparoscopic adjustable gastric band (LAGB), are currently the only effective treatments for morbid obesity. The LAGB exerts its effects on satiety, reducing food intake and body weight by the modulation of both neural and hormonal responses, with the latter involving an elevation of meal-related levels of glucagon-like peptide-1 (GLP-1) and peptide YY (PYY) [88]. The brain, mainly the hypothalamus, is the organ responsible for maintaining the balance between food intake and energy expenditure by receiving peripheral signals from adipose tissue (adipose signals) and responding to them. Several neuropeptides are involved in this process, such as neuropeptide Y (NPY), which increases food consumption and decreases energy expenditure. NPY, acting through specific receptors, plays an important role in several physiological functions, including cardiovascular homeostasis and regulation of sympathetic nervous system activity. It mediates stress-induced obesity in adult male mice by activating its Y2 receptor (Y2R) in visceral adipose tissue. In fact, the expression of NPY is correlated with body weight changes, rather than with the presence of type 2 diabetes [89].

Chemerin is a recently identified adipokine that is highly expressed in liver and adipose tissue and is associated with adiposity, insulin resistance, MetS risk factors, and degree of nonalcoholic fatty liver. Importantly, chemerin is thought to regulate adipogenesis and metabolic homeostasis in murine and human adipocytes. Additionally, it modulates the innate immune system through its binding to the orphan G-protein coupled receptor chemokine-like receptor 1. It also modulates chemotaxis of immature dendritic cells and macrophages. Recent studies have associated chemerin with several inflammatory markers in obesity and T2D. Thus, chemerin is considered as a candidate in linking inflammation to obesity-related diseases [90].

Weight gain and the appearance of insulin resistance go hand in hand and are thought to be caused by an abnormal adipokine release by visceral fat. Adipocytes release retinol binding protein 4 *(*RBP4) and visfatin, and their plasma levels are elevated in individuals with abdominal obesity. The observation that RBP4 induces insulin resistance by interfering with insulin receptor substrate 1 adds to the list of plasma abnormalities that link obesity with the development of T2D. At levels found *in vivo* these adipokines interfere with insulin receptor substrate 1 regulation (short contact) and induce its degradation (prolonged contact) [91]. Another treatment for obesity consists of behavioral weight management interventions, which consistently produce 8% to 10% reductions in body weight, although most subjects regain weight after treatment ends. One strategy for extending the effects of behavioral interventions has been the provision of extended care, which is a viable and efficacious solution to addressing the long-term maintenance of lost weight. Given the chronic disease nature of obesity, extended care may be necessary for long-term health benefits [92].

About the modulation of both neural and hormonal responses, periodontitis has also been associated with preterm birth. For many years, the incidence of preterm birth has not decreased in developed countries despite the promotion of public health programs. Many risk factors have been identified, including ethnicity, age, tobacco, and infection. However, the causes of almost 50% of preterm births remain unknown. As periodontal diseases are highly prevalent and negatively influence general health, worsening the incidence of CVD and diabetes, they have also been suspected to increase the rate of preterm birth. However, data on this topic remain contradictory. Physicians/obstetricians can identify women at risk of preterm birth and refer these patients to dentists for periodontal examination and treatment to limit adverse pregnancy outcomes [93].

## Hypertension and periodontitis

Hypertensives suffering from MetS show increased oxidative stress and compromised antioxidant activity in plasma and cells [94,95]. In addition, obesity and overweight are strictly related to hypertension. In fact, weight loss determines a diminished blood pressure independent from sodic diet [95]. Moreover, hyperglycemia and hypertension are strictly related. Hyperglycemia provokes an increased stimulation of a sympathetic nervous system that causes vasoconstriction and increased sodium reabsorption with consequent water attraction and insurgence of hypertension, which damages the endothelium integrity of vessels [96].

Augmented endothelial permeability allows the passage of lipoproteins and platelet-derived growth factors (PDGF), which give rise to the proliferation of muscular smooth cells in the intima, which occludes vessel lumen and causes embolia, hypoxia, and consequent cellular death [97].

It also seems that periodontitis can influence some types of hypertension [98]. Several studies have taken into consideration the relationship between hypertension and periodontitis, although an association between periodontal disease measures and incident hypertension in cohort studies has not yet been evidenced. In a sample of 31,543 participants of the Health Professionals’ Follow-Up Study, based on a prospective cohort of 40- to 75-year-old men at baseline, with no prior hypertension history and complete baseline information on oral health, an incidence of 10,828 cases of hypertension over 20 years of follow-up was identified, with no significant association between incident hypertension and periodontal disease [99].

In a study population of 182 adults, a multivariate analysis showed no association between severe periodontitis and hypertension history (OR = 0.99; 95% CI: 0.40-2.48). Severe periodontitis was associated with high blood pressure, with an OR of 2.93 (95% CI: 1.25-6.84) after adjusting for age, gender, smoking, and binge drinking. This association was stronger when restricted to those with hypertension or taking antihypertensive medications (OR = 4.20; 95% CI: 1.28-13.80), suggesting that periodontitis may contribute to poor blood pressure control among older adults [100].

Although statistical evidence is lacking, a clinical relation between high blood pressure and aggressive periodontitis has been deduced, as patients with poor oral hygiene have higher blood pressure problems than do healthy subjects with good oral hygiene condition [99]. Regarding the biological mechanism of this relationship, a recent study evaluated endothelial function in patients with periodontitis. Circulating levels of CRP and IL-6 were significantly higher in the periodontitis subjects with hypertension, than in the control group. Periodontal therapy seems to reduce serum concentrations of CRP and IL-6 [101].

## Metabolic syndrome and periodontitis

As already mentioned, MetS is a syndrome characterized by several signs that together seriously compromise the health of an individual. It is clear that the common denominator of the member pathologies of MetS is oxidative stress and the consequent hyperinflammation that primes chain interactions and leads to grave systemic complications, such as CVD, or local complications, such as periodontitis.

MetS allows a pro-oxidative state in periodontal tissue, altering antioxidant defense mechanisms. This adversely affects tissular response against bacterial plaque attack. On the contrary, periodontitis, being a great source of oxidative markers, promotes the onset of insulin resistance and MetS in a vicious circle [102]. Chronic inflammation during old age periodontitis causes increased neutrophil defense activity, which involves increased oxidative activity, resulting in peroxidation and oxidative stress. In fact, both MetS and periodontitis show increased serum rates of oxidative stress markers [103,104].

Regarding the oxidative stress markers found in periodontitis, individuals with periodontal disease exhibit a significant increase in the activities of oxidative stress markers. The increase in glutathione peroxidase may represent possible antioxidant compensation in detoxification reactions of organic peroxides produced during oxidative stress in gingival tissue. Since glutathione S-transferase (GST) has a direct role in the neutralization of hydroperoxides derived from the lipoperoxidation processes, increases in GST activities are probably related to the oxidative stress caused by the periodontal inflammatory process. GST comprises a group of enzymes that are also able to detoxify a variety of compounds, including xenobiotics derived from pathogenic microorganisms. Hence, increases in GST activities are excellent indicators of endogenous detoxification from exogenous sources. Myeloperoxidase activity in gingival tissue has shown a significant increase in patients with periodontal disease when compared with controls: this seems indicative of a chronic inflammatory process also reflected at a systemic level. A significant increase in oxidized glutathione (GSSG) concentrations has been detected in periodontitis patients, which is a clear biomarker of oxidative stress detected in inflammatory processes linked to periodontitis. Consistent with the results for GSSG, tissue lipoperoxidation, measured as thiobarbituric acid reactive substances, seems to increase in the gingival tissue of periodontitis [105]. Periodontal diseases seem related to pathologies and conditions characterized by high oxidative stress and by the presence of AGE, such as diabetes and physiologic aging. AGEs are able to favor chemotaxis and the production of proinflammatory mediators, to inhibit fibroblasts and osteoblasts, and to accelerate periodontal damage directly or binding their receptors RAGE [106]. Periodontitis is strictly correlated to hyperglycemia; in fact, it is also considered the sixth complication of diabetes mellitus [61]. Predialysis and hemodialysis in chronic kidney diseases are also associated with a higher prevalence of severe periodontitis compared with healthy individuals.

Chronic kidney failure is a clinical syndrome due to the slow, progressive, and irreversible loss of the glomerular filtration rate, and may be associated with several oral manifestations, such as xerostomia, uremic stomatitis, and periodontitis, diagnosed as clinical attachment loss.

Recent studies have shown an association between high levels of CRP and IL-6 and periodontitis, an association that decreases after periodontal treatment. Due to this association with the systemic inflammatory response, chronic periodontitis has recently been included as a nontraditional risk factor for chronic kidney failure [107].

In synthesis, metabolic alterations related to MetS component diseases cause an augmented response to bacterial plaque, which favors periodontitis insurgence.

It has been pointed in many studies out how periodontal treatment can reduce inflammatory mediators related to endothelial and cardio-circulatory dysfunctions [108]. A very recent work reported a real relationship between periodontitis and MetS, especially in women, while abdominal obesity was the largest contributory factor in both genders [109]. On the contrary, another new work about MetS and periodontal diseases and caries did not find a strong association between MetS and periodontal infections [110].

## Odontologic management of metabolic patients

Dentists play a key role in the precocious diagnosis of MetS and its local complications such as periodontitis.

### The diet

The capability of an aliment to raise the level of insulin in the blood, compared with a reference food such as glucose or with bread, is known as the glycemic index (GI) [111,112]. It seems that the greater the consumption of foods with higher GI, the wider will be the risk of developing insulin resistance, endothelial dysfunction, and CVD. In fact, taking foods with high GI results in the quick increase of glycemia and, consequently, in insulinemia in the attempt to restore glycemia; the secretion of insulin continues in spite of reestablished euglycemia, so as to conduce to a hypoglycemic state [113].

Hypoglycemia causes the secretion of counter-regulatory hormones, such as corticosteroids, glucagon, and epinephrine, with the consequent reduction of lipolysis, glycogenolysis, fatty-acid release, and vasoconstriction that can bring hypertension [114]. Repeating this process may lead to β-cell damage and T2D. A poor diet of foods with high GI is basilar to reduce at least the risk factor. Several studies have demonstrated the relationship between the intake of foods with high GI and MetS [115]. It is ideal to consume nutrient-rich, high-fiber food such as fruits and vegetables to maintain good health [116].

### Good oral health

Maintaining good oral health is fundamental for individuals who suffer from MetS and have a tendency to develop CVD. Progressive loss of teeth produces a variation of diet, with an increased intake of foods with great caloric rate, saturated fats, trans fatty acids, and cholesterol, and less intake of fruits and vegetables rich in vitamins and fibers, folate, and potassium [117]. The use of partial or total removable prosthesis does not seem to cover the masticatory efficacy of natural teeth [118], but fixed dental prosthetic devices and prosthetic overimplants seem to improve dietary practices [119]. Drugs used in patients suffering from MetS may give rise to oral collateral effects and could interact with drugs prescribed in dentistry [120].

Sibutramine, used in weight control, may cause hypertension and tachycardia, and if associated with opioids, may provoke serotonin syndrome with confusion, palpitations, and loss of consciousness; the concomitant use of erythromycin and clarithromycin could lead to toxicity. Ace inhibitors, if prescribed in association with nonsteroidal anti-inflammatory agents, may be inhibited in their activity. Statin medications in association with erythromycin and clarithromycin could lead to renal failure [97]. As diabetes is a member of MetS, dentists may evaluate glycemic value before starting dental therapeutic measures [121].

The oral complications of diabetes are candidiasis, xerostomia, burning mouth syndrome, gingivitis, oral acute infections, and, clearly, periodontal diseases – all diseases treated with dentistry [122]. Therefore, the role of dentists in the diagnosis, therapy, and management of metabolic patients is fundamental [123], and an improvement of collaboration among dentists, cardiologists, endocrinologists, and dietists is needed to promote the multidisciplinary therapeutic approach to this syndrome.

## Conclusions

In this review, we tried to explain how various pathologies associated with MetS can be related to and favor the onset of CVD and, especially, periodontitis. Oxidative stress seems to be the chief suspect in ethiopathogenesis of periodontal disease; for this, the use of drugs with antioxidative activity or anti-AGE is the subject of research. Chemical agents such as pyridoxamine, metformin, and nefedipine, with their antioxidative ability, could be used. Other anti-AGE drugs are modified tetracyclines, such as doxycycline, which have anti-inflammatory and antioxidative effects. However, this argument needs more clarity, and the search for answers goes encouraged. As we await more results, we can increase prevention in at-risk individuals by advising lifestyle changes and prescribing a balanced diet to control body weight, hyperlipidemia, and hypertension; advising a stop to smoking and the maintenance good oral hygiene in periodontal therapy; and finally, establishing pharmacological and eating control of diabetes.

The role of dentists in the diagnosis, therapy, and management of metabolic patients is fundamental, but an improvement of collaboration among dentists, cardiologists, endocrinologists, dietists, etc., is needed.

## Competing interests

The authors declare that they have no competing interests.

## Authors’ contributions

EM coordinated the revision of literature; AM described main results; LP revised the literature; SM coordinated the introduction; RG coordinated the discussion paragraph; STetè revised the bibliography,; AB reviewed the literature suggested by the reviewers; STecco conceived the study, analyzed the data, coordinated the discussion, and draft the manuscript; GM conceived the study and coordinated the analysis of data. All authors read and approved the final manuscript.
